# Implementation of cardiovascular disease prevention in primary health care: enhancing understanding using normalisation process theory

**DOI:** 10.1186/s12875-017-0580-x

**Published:** 2017-02-24

**Authors:** Nerida Volker, Lauren T. Williams, Rachel C. Davey, Thomas Cochrane, Tanya Clancy

**Affiliations:** 1 Health Research Institute, Centre for Research & Action in Public Health, University of Canberra, College Road, Bruce, Canberra, ACT 2601 Australia; 2 Menzies Health Institute of Queensland, Griffith University, Parklands Drive, Southport, QLD 4222 Australia

**Keywords:** Practice nurse, Health coaching, Lifestyle modification, Preventative health

## Abstract

**Background:**

The reorientation of primary health care towards prevention is fundamental to addressing the rising burden of chronic disease. However, in Australia, cardiovascular disease prevention practice in primary health care is not generally consistent with existing guidelines. The Model for Prevention study was a whole-of-system cardiovascular disease prevention intervention, with one component being enhanced lifestyle modification support and addition of a health coaching service in the general practice setting. To determine the feasibility of translating intervention outcomes into real world practice, implementation work done by stakeholders was examined using Normalisation Process Theory as a framework.

**Methods:**

Data was collected through interviews with 40 intervention participants and included general practitioners, practice nurses, practice managers, lifestyle advisors and participants. Data analysis was informed by normalisation process theory constructs.

**Results:**

Stakeholders were in agreement that, while prevention is a key function of general practice, it was not their usual work. There were varying levels of engagement with the intervention by practice staff due to staff interest, capacity and turnover, but most staff reconfigured their work for required activities. The Lifestyle Advisors believed staff had varied levels of interest in and understanding of, their service, but most staff felt their role was useful. Patients expanded their existing relationships with their general practice, and most achieved their lifestyle modification goals.

While the study highlighted the complex nature of the change required, many of the new or enhanced processes implemented as part of the intervention could be scaled up to improve the systems approach to prevention. Overcoming the barriers to change, such as the perception of CVD prevention as a ‘hard sell’, is going to rely on improving the value proposition for all stakeholders.

**Conclusions:**

The study provided a detailed understanding of the work required to implement a complex cardiovascular disease prevention intervention within general practice. The findings highlighted the need for multiple strategies that engage all stakeholders. Normalisation process theory was a useful framework for guiding change implementation.

**Electronic supplementary material:**

The online version of this article (doi:10.1186/s12875-017-0580-x) contains supplementary material, which is available to authorized users.

## Background

Cardiovascular disease (CVD) was the leading cause of death in Australia in 2011 [1]. Primary health care has an important role in supporting CVD prevention, however prevention-orientated activities are not routinely undertaken in Australian general practice [2, 3]. While the need for more heath promoting health systems has long been recognised, attempts to reorientate health services towards prevention have proven highly resistant to change [4]. The need for greater expertise in how to implement CVD prevention strategies in practice has been identified as key to addressing the CVD burden worldwide [5].

The Model for Prevention study (MoFoP) is a case study exploration of a whole-of-system CVD prevention intervention framed by the Expanded Chronic Care Model (ECCM) [6].

The ECCM provides an evidence-based approach to health system redesign for prevention and management of chronic disease integrating the Chronic Care Model and the five action areas from the Ottawa Charter for Health Promotion [7]. The intervention was of 12 months duration, with strategies including improvement of clinical and community information systems, support for health practitioner decision making for CVD risk management, provision of a health coaching service to support patients to develop lifestyle modification skills and health system redesign to provide greater health behaviour change support across the general practice setting.

Patients from six general practices in the Australian Capital Territory (ACT) identified via existing clinical data as being at high risk for CVD disease were provided with enhanced risk management support, including access to a Lifestyle Advisor service (health coach) for up to 12 months (average of four sessions). The intervention also included strategies to build the capacity of community-based lifestyle modification services to support patients in the community setting.

Interventions to address chronic disease are complex and, while guidelines-based care has been shown to be effective in very controlled situations, translating these outcomes into real world practice has proven difficult to sustain. In their review of Chronic Care Model-framed interventions which aim to improve chronic disease outcomes, Kadu and Stolee found the need for more research focused on understanding the inner settings of organisations, including the characteristics of the work of individual practitioners, in order to better understand how to achieve and sustain positive outcomes [8]. One of the main aims of the MoFoP study was to focus on the feasibility of embedding the intervention approach into real world practice, both in the general practice and community setting. The community setting aspects of the study have been reported elsewhere [9].

This paper focuses on the aspects of the intervention that occurred within general practice involving general practitioners (GP), practice nurses (PN), practice management (PM), lifestyle advisors (LA) and patients who participated in the intervention (P). To make sense of the social and organisational aspects of the intervention Normalisation Process Theory (NPT) was chosen as a tool to frame the analysis. NPT is a mid-level theory developed to understand and evaluate the processes by which complex interventions are embedded into routine practice [10]. The theory takes a whole-of-system perspective, which aligned well with the intervention design. NPT has also been used as a tool for assessing the suitability of trial approaches or providing information to optimise trials [11].

## Methods

### Data collection

The study used a qualitative design employing semi-structure interviews. The interviews were conducted with staff of all the six practices, who delivered the CVD prevention intervention. All staff members were sent individual invitations by the research team to participate in the interviews. Additionally, all patients who participated in the intervention (*n* = 30) and both Lifestyle Advisors were sent invitations to be interviewed. Semi-structured interviews were used instead of focus groups due to the nature of the general practice environment, which makes it difficult for groups to get together at one time. Interviews also provide a degree of anonymity for staff members who were often employees or junior staff of other interviewees. Topic guides for practice staff and LAs were informed by NPT constructs. These constructs help to explain the work involved in embedding interventions into routine practice. This includes making meaning and sense of the intervention (coherence), committing to and engaging with the intervention (cognitive participation), delivering the intervention (collective action) and reflecting and appraising the intervention approach (reflective monitoring).

Topic guides for the interviews with patients were developed in very early stages of the research and contained evaluation questions not directly informed by NPT. The decision was made to include patient data in this analysis given the identified need to include service user evaluation in the NPT literature [12]. All topic guides were pilot tested before data collection commenced. Interview questions for all stakeholders are provided in Additional file 1.

Purposive sampling was used to ensure representation from a range of practice staff across all of the intervention general practices. Practice staff were invited to participate via a letter from the researcher, which was distributed by practice management. Patients and LAs were also invited by letter to participate after the completion of the intervention. Interviews with general practice staff were conducted mostly on site at practices and lasted between 20 and 60 min. Interviews were conducted individually rather than in a group due to sensitivity to employee/employer relationships which may have impacted on the ability of some staff to answer questions honestly. Patients were interviewed face to face at their general practice or by phone (for their convenience) and lasted between 30 and 60 min. LAs were interviewed face to face at the office of the interviewer and these interviews lasted around 60 min. Interviews were conducted between December 2013 and July 2014. All interviews were conducted by the first author, audio taped and transcribed. Ethical approval for the study was obtained from University of Canberra Human Research Ethics Committee (Project number 11–141).

A description of each of the stakeholder groups is outlined in Table 1.

**Table 1:** Description of key stakeholder groups

Title	Description of key stakeholder groups
General Practitioner	GPs are medical practitioners who provide primary, comprehensive and continuing care to patients and their families within the community. General practice is a medical speciality. Entry to the speciality may be achieved by admission to Fellowship of the Royal Australian College of General Practice [22].
Practice Manager	PMs perform all or some of the practice management tasks in a healthcare setting [23].
Practice Nurse	PNs are registered or enrolled nurses who are employed by, or whose services are otherwise retained, by a general practice [24].
Lifestyle Advisor	LAs (health coaches) perform a relatively new care extender role helping patients gain the knowledge, skills, tools and confidence to become active participants in their own care so that they can reach their self-identified health goals [25]. LAs were fitness professionals provided with additional training in the Health Change Australia approach [26].
Intervention Participant (patient)	Ps were those patients who responded to the recall and participated in the intervention.

### Data analysis

The study used NPT constructs to frame the data analysis. May and colleagues proposed that NPT could structure the way that qualitative data is coded, analysed and understood [13]. In this instance, the four constructs (and components) were used to code the data in line with Strauss and Corbin’s single coding approach [14]. Drawing on the work of Murray, Blakeman, and Gallacher, a NPT informed coding framework was developed and interview transcripts were coded against the framework [15, 16]. The framework is described at Table 2.

**Table 2:** Coding framework for analysis

Coherence (sense making work)	Cognitive participation (Relationship work)	Collective action (Enacting work)	Reflexive monitoring (Appraising work)
Differentiation	Enrolment	Interactional workability	Reconfiguration
Were staff and patients clear on their roles regarding the intervention? Were staff and patients clear on other’s roles regarding the intervention?	Did staff engage with other staff around the intervention? Did patients engage with the practice for the intervention? Who initiated the engagement? Who did and who didn’t ‘buy-in’ to the intervention?	How was the intervention enacted by staff, Lifestyle Advisors and patients? How did the intervention fit with existing work of all stakeholders? How did patients, staff and lifestyle advisors adapt to the introduction of the intervention? What effect did the intervention have on usual practice?	Has the approach to CVD prevention in the practices been adapted based on the intervention experience? If so, how? Has the patients approach to their CVD risk changed based on the intervention experience? If so, how?
Individual specification	Initiation	Relational integration	Individual appraisal
Did the staff and patients know what the intervention was? Was the intervention easy for the staff and patients to describe? What benefits did the intervention bring, and to whom?	Who was engaged in the intervention? What organisational skills did staff use to contribute to the intervention? Were staff prepared to invest time, energy and work to the intervention? If so, what did they do? Were patients prepared to invest time, energy and work into the intervention? If so what did they do? What organisation skills did they use?	How did the intervention affect trust and confidence between all parties (i.e. patients, staff, lifestyle advisors)? How did the intervention affect the relationships between all stakeholders? How did all stakeholders work to maintain relationships?	Was it clear to staff and patients what effects the intervention has had? Did patients and staff make efforts to reflect on and appraise the intervention? If so, how? Has appraisal work informed whether the intervention was advantageous for patients and staff?
Communal specification	Activation	Skill set workability	Communal appraisal
Did the staff have a shared sense of purpose around the intervention? Did staff and patients have a shared sense of purpose around the intervention? Who thought the intervention was a good idea? Who did not? Were the benefits of the intervention valued by all patients and all staff? Did the intervention fit with the overall goals and activity of the general practice?	Did the patients and staff undertake work to arrange a shared contribution to the implementing the intervention? If so, what is this work? How did the intervention feature in practice meetings? How did the practice communicate with patients about the intervention?	How was the intervention work distributed within the patients and staff? What impact did the introduction of the intervention have on the distribution/division of labour, resources, power and responsibility? Was the work for the intervention devolved to others not usually doing the work? If so, how and for what reason? Was there alignment in the intervention approach throughout the patients and staff? Did the introduction of the intervention alter the awareness of the work done by other members within a practice team?	How did patients and staff know that the intervention approach is being carried out? Did staff and patients contribute/share feedback about the intervention with others? If so, what was discussed? Has appraisal work informed whether the intervention approach is advantageous for patients and staff?
Internalisation	Legitimation	Contextual integration	Systematisation
Was there an understanding by staff of how to learn to implement the intervention approach? Did the patients relate their CVD risk and their need to seek support to the intervention? Did staff have the time to learn to understand and carry out the intervention approach?	Was there work undertaken to ensure that participating in the intervention was viewed by patients and staff as the right thing to do? If so, what was this work? Did staff have the permission to use the intervention approach?	How was the intervention resourced? Did patients bring their own resources to the intervention? Could they integrate lifestyle changes into their everyday life? Are tasks compatible with existing work practices? Was the intervention linked to, and resourced through, organisational structures (e.g. clinical information systems, decision support tools)? How did the intervention affect the relationship with existing structures?	Has the practice developed ways of keeping patients and staff up to date with best practice approaches to CVD prevention?

A subset (20%) of transcripts were coded independently by a second researcher and then compared and discussed to ensure consistent coding against NPT constructs. The data generally aligned with the constructs and, where data did not fit, it was coded as “other”. Data handling was facilitated using NVivo 10 software. After coding, narratives were developed under each of the constructs.

## Results

There were 40 face to face interviews conducted with participants in all six general practices involved in the MoFoP study. Interviews were held with 11 General Practitioners (GPs) (26% of GP participants), 12 Practice Nurses (PNs) (75% of PN participants), six Practice Managers (PMs) (100% of PM participants), two LAs (100% of LA participants) and nine patients (30% of patient participants). Characteristics of interview participants are detailed in Table 3.

**Table 3:** Characteristics of stakeholders interviewed

PNs	Years as PN	PMs	Years as PM	GPs	Years as GP	LAs	Years in lifestyle modification	Patients (P)	Age in years
PN1	3	PM1	19	GP1	N/A	LA1	27	P1	69
PN2	6	PM2	4	GP2	<1	LA2	27	P2	72
PN3	6	PM3	16	GP3	25			P3	66
PN4	10	PM4	8	GP4	1			P4	66
PN5	1	PM5	1.5	GP5	25			P5	69
PN6	7.5	PM6	18	GP6	1			P6	73
PN7	1.5			GP7	8			P7	65
PN8	1			GP8	1			P8	70
PN9	1			GP9	2			P9	75
PN10	0.5			GP10	20				
PN11	3			GP11	25				
PN12	5								

While male and female practice staff members were interviewed, gender is not reported for each category to maintain confidentiality of individuals. All patients interviewed were male, consistent with the total MoFoP intervention population, where all but one participating patient was male.

The stakeholder interviews provided a rich description of the processes of implementation of the MoFoP intervention. After the data were analysed using the NPT coding framework, narratives were developed for each of the four NPT constructs. These narratives were summarised and are presented below with illustrative quotes. While not all elements within each construct had the same density of coded information, overall most stakeholders could comment across most of the NPT constructs. The quotes are attributed to participants by professional grouping, years working in that role, and age for patients.

### Making sense of the intervention

For the general practices and their patients, the intervention was the first time they had taken a systematic approach to identification and management of CVD risk.

The prevention-orientated service was considered by practice staff to be important for the health of their patients and an important function of general practice, as one GP stated:

*I feel this is what general practice is about. We are here to prevent health problems and manage patients.* (GP11, 25 years as a GP).

While practice staff saw the intervention approach to be consistent with the goals of general practice, most agreed it was different from their everyday experience, which focused on responding to acute illness. As one PN put it, “*Normally it is about treating; this was about prevention*” (PN7, 1 year as a PN).

The patients who participated in the intervention also agreed that preventing CVD was important and they expected their general practice to have a role in supporting them to reduce their risk. Most reported that they had existing risk factors for CVD and felt the intervention was therefore relevant to them. However, not all participants were convinced that they were at high risk and for one patient the recall letter was totally unexpected.

He said, “*I think the word I would use is bemused. I didn’t know, or didn’t really suspect, that I might have been at high risk*” (P4, 66 years old).

### Stakeholder investments in the intervention

While the six general practices volunteered to be involved in the intervention, the degree of commitment and engagement varied across the practices, between practice staff groups and for patients in each of the different practices.

One PN noted that in their practice “*there was great support from management, but I think the clinicians (GPs) had varying levels of commitment to the project.*” (PN 2, 6 years as a PN).

In another practice a strong interest in CVD prevention by one particular staff member led to a high level of engagement with the intervention. A GP from this practice noted, “*Having a nurse with a background in cardiovascular disease meant that we weren’t going to let anything cardiovascular pass us by*” (GP 1, 20 years as a GP). While many practice staff reported that they participated in the intervention in the interests of their patients, others undertook activities because they were expected to as part of their employment. As one GP said “*I did what I was told*” (GP3, 25 years as a GP). Both LAs also felt that there were varying levels of support for the intervention across practices with one LA stating that*,* “*I would probably say one of the four (GPs) was committed*” (LA2, 27 years in lifestyle modification).

Communication with practice staff regarding the delivery of the intervention was generally good. However, staff turnover in all practices meant that as the intervention progressed some practitioners were not informed of their required activities. One GP noted that “*I think the doctors who came along after the intervention had started did not know what was involved…they did not go to the talks*” (GP1, 20 years as a GP).

The patients interviewed had all made the effort to participate in the intervention by reading the provided information on CVD risk, making an appointment to see their GP, and in some cases, having a blood test to update lipid levels. Most patients considered the intervention to be a normal extension of care from their general practice. They had established relationships with the practice and its staff and assumed that participation would be beneficial. As one patient said “*It seemed ordinary, in a sense that this practice does look after me*” (P5, 69 years old).

### Stakeholders work to enact the intervention

While all practices reported following the intervention protocol, the size of the practice, skills and preferences of the workforce, and the business model led to the work of the intervention being configured in a range of ways. These ranged from a nurse led approach to the intervention, with limited input by the GP, to non-participation where individual GPs in a practice would not allow their patients to be recalled because they did not receive a direct incentive payment. One GP explained:

*It depends on how the practice is set up and what your nursing staff are like. I suppose also how you like to do things as a GP as well. If you like to maintain that relationship and do the education yourself rather than pass the buck to whoever it may be.* (GP9, 2 years as a GP)

Successful implementation of the intervention relied on administration staff undertaking a wide range of tasks, including many considered central to achieving a systems approach. While clinicians were responsible for recording most of the clinical and demographic data, administration staff took the lead in most practices in educating and reminding staff about the need to enter the key demographic and CVD risk factor data and how to enter this data correctly. They used their usual practice communication strategies such as staff meetings and some novel approaches, for example, messages on the back of staff toilet doors. One PM recounted her approach, *“We were sticking little stickers* [Post It notes] *all over the GP’s computers saying, “please do this or please do that”.* (PM4, 8 years as a PM).

Other tasks that were central to successful implementation were related to navigating patients through the intervention processes, such as responding appropriately to phone calls about the recall visits. When administration staff failed to perform these tasks the practice experienced difficulties communicating with eligible patients. One patient recalled his experience of contacting his practice and the receptionist not being fully aware of her role in the intervention.

*I had to ring up the practice and tell them that I wanted an appointment for this program. So I did that and they made me feel that I was stupid…why did I need this appointment she said.* (P8, 70 years old).

All GPs reported that the intervention did not add a significant workload and they were happy to refer patients to the LA service. They frequently commented that it was beneficial to give patients access to a broader range of team members who could spend more time with them on lifestyle modification issues. As one GP said:

*I can only do so much for this patient because I have 15 minutes … so that team-based model… I think the program got that team approach.* (GP1, 20 years as a GP).

For the PNs the intervention activities led to a greater consultation role, which most of them enjoyed. This work differed from their usual task-orientation and provided opportunities to build deeper relationships with patients.

One practice nurse said, “*There is not a lot of relationship building (usually). When talking about prevention, we need to build relationships, and be in it for the long haul*” (PN2, 6 years as a PN).

The LAs were highly motivated to develop good working relationships with the GPs from the beginning of the intervention. While they reported to be disappointed that the contact was generally restricted to written communication, they did feel that being present at the practice made their service more credible with the clinical staff.

One LA said “*they had more confidence in what we do because we were actually in the medical centre.*” (LA1, 27 years working in lifestyle modification).

While the intervention introduced an LA as a new health care provider to the usual practice based care team, the patients did not feel that their relationship with their GP or other practice staff was diminished. As one participant stated, “*I don’t think he thought I was being poached away.*” (P 3, 66 years).

Most found that the health coaching approach used by the LAs was non-judgemental, allowing them to be honest about areas they were willing to change and supported in achieving the goals they set for themselves. One patient outlined how the process worked for them:

*Basically what happened is I told her what I wanted to do, she listened and I took over. She steered me in another direction sometimes or pointed something out I might need.* (P7, 65 years old).

### Stakeholder appraisal of the intervention

All those interviewed considered the intervention to be worthwhile at some level. Most patients made positive changes to their lifestyle as a result of the intervention. One patient had good outcomes for issues that had previously been resistant to change. He said:

*I have certainly reduced smoking and alcohol. I have been able to put alcohol free days into my program which I found difficult before* (P1, 69 years old).

Practice staff and LAs recalled positive health and wellbeing outcomes for patients and the practice and systems level changes achieved. These changes included both practice and opportunistic risk factor data captured for enhanced CVD risk assessment and management. As explained by one of the PNs interviewed:

*Since the program was introduced I think it improved our collection of data, when patients come now all patients who come through the treatment room get height, weight, blood pressure, allergies and smoking assessment* (PN12, 5 years as a PN).

The key role of administrative staff improving the systems response to CVD prevention was also recognised during the study. One experienced PM noted that “*Since my involvement with this project I train my staff that are sending out the recall letter… so when a patient calls and makes an appointment they know exactly what to tell them*” (PM6, 18 years as a PM).

Some staff believed the intervention resulted in a greater awareness by GPs of the importance of managing CVD risk and an increased number of CVD risk assessments being conducted. One PN reported that in her practice, CVD risk assessment had increased, stating that “*Doctors are doing a lot more cardiovascular assessments…they are actually doing them!*” (PN2, 6 years as a PN). There were staff in every practice that identified barriers to them reorganising practice to align with the intervention approach. While practice nurses were interested in an expanded role, many felt underprepared to address lifestyle modification and felt they needed more training in the area. This was highlighted by one nurse who said, “*I haven’t been trained for any of this, it has all been a learning curve for me*” (PN6, 7 years as a PN). Current financing models, time pressures and practical issues such as poorly integrated clinical software were also considered issues to be addressed if they were to become a more prevention-orientated practice. One GP was very clear about this, stating that:

*Anything that puts more work on the staff or the doctors is unrealistic… If there’s no incentive for us, at least make it that simple that I don’t have to invest extra work into it.* (GP10, 20 years as a GP).

When reflecting back on the intervention, almost all practice staff identified the difficulty of achieving sustained lifestyle modification itself as a major impediment to providing prevention-orientated services. They also considered these challenges would be exacerbated if there were out of pocket costs for patients.

*It’s a hard sell and that is why it should be free.* (PN7, 1 year as a PN).

## Discussion

The study examined the work required by general practice staff, Lifestyle Advisers and patients to implement CVD prevention in primary care consistent with existing evidence-based practice guidelines [17]. The findings provide insight into the feasibility of intervention approach being embedded into real world practice. There were many aspects of the existing general practice system that could be supported, some with very small investment, to achieve and sustain more health promoting general practice setting. However, significant barriers to system change did exist, such as the design of current funding and the challenging nature of lifestyle modification, making it difficult for practitioners, and their patients, to move away from their usual practice.

While usual practice did not normally include prevention-orientated activities, practice staff felt that prevention-orientated care was part of their role and that of general practice. Patients agreed that CVD prevention was important, important to their own health and they expected their General Practice to support them to reduce their risk of CVD. To enhance the likelihood of the intervention approach being adopted, the study outcomes highlight the need for an increased focus on strategies that build relationships across general practice. Mazza and colleagues found that for patients, having trust and a good rapport with a GP encouraged them to participate in prevention-orientated care [18]. Consistent with this finding, most patients in the intervention identified strongly with the General Practice or their particular GP. The study outcomes also emphasised the value of nurturing relationships between patients and other members of the practice team. Practice Nurses were open to building these relationships and developing more comprehensive clinical roles. The administration staff also had an important role in engaging patients into the intervention particularly in areas related to coherence, supporting patients to see their general practice as interested in prevention and cognitive participation, getting the multiple internal and external relationships to work for the intervention and making it easy for patients to engage in the new activities. Greater recognition of the importance of this workforce and the development more specific strategies to support their role, should be a feature of future interventions.

Redistribution of the work undertaken by general practice staff to support patients in health behaviour change was an important element of the intervention approach. This included the adoption of new or enhanced tasks related to health information quality, CVD risk assessment and behaviour change support for the practice staff. There was also the integration of a new primary health care workforce in the Lifestyle Advisors. The GPs were generally happy to undertake small additions to practice to deliver the intervention and to transfer more lengthy discussions on lifestyle modification to the LA. Most PNs welcomed the opportunity to expand their role, but found lifestyle modification a challenging area of practice because they had had little previous training or experience in the area. Limitations in health behaviour change skills of nurses working in primary health care and has been previously identified as a barrier to PN’s more active engagement in chronic disease prevention [19]. Ongoing professional development, particularly using an academic detailing approach that offers tailored on-site support for the different needs of individual staff and practices, is needed to build the competence and confidence of this workforce in this new role.

The positioning of a health coaching service in a primary care practice is a relatively new innovation. While most patients and practice staff reported positive outcomes from the LAs service, the LAs themselves perceived varying levels of interest, or as they put it “curiosity” about their service, particularly by practice staff. Liddy and colleagues found that it does take time for practices to adjust to having this new role in place and that a poor understanding of health coaching by other practice staff can limit its effectiveness [20]. Therefore the addition of this new workforce has potential to make prevention-orientated services easier to deliver in the general practice setting, but it will need to be supported over an extended period to allow the potential benefits to be fully recognised. To continue this role would be an area requiring a new source of recurrent funding, as in the current intervention these positions were fully supported with short term project funds.

The need for more recurrent funding for prevention activities and the challenging nature of lifestyle modification were identified as fundamental barriers to the intervention approach being adopted as usual practice. The lack of adequate funding, funding along with limited time and other competing priorities have been found by other researchers to be barriers to lifestyle modification in general practice [21]. This ambivalence remains a fundamental barrier to prevention-orientated general practice, with the benefits of moving to delivery of more prevention services over remaining with the status quo, not yet compelling for the practices involved in the study. However, if positive patient health and practice system outcomes could be maintained, even at some level, over time the experience of CVD prevention could improve and help to build the value proposition for prevention-orientated services. The need to build the value proposition for prevention was also emphasised by the community-based lifestyle modification providers in their attempt to operate viable businesses [9]. Until prevention-orientated services hold greater value for everyone and attract the collective action and subsequent advocacy for policy and funding reform, it is unlikely there will be the community and political will required to achieve change.

The study demonstrated the utility of exploring implementation processes and the work required at an individual and organisational level to see translation of evidence-based CVD prevention practice into everyday general practice. It also showed the benefit of using NPT as a tool for examining implementation of a prevention-orientated activity. It allowed the researchers to ‘think through’ the data in a structured way and highlighted the work required by all stakeholders to implement such a complex intervention. Much of this information was unlikely to have been captured systematically in other ways. In particular, it helped to expose the ‘hidden work’ that needs to occur to create health promoting systems.

The limitations of the study relate to the sample and the timing of the interviews. The study took place in a single city with a generally high standard of living. The sample of practice staff included only those willing to be interviewed, who may have been more interested in the intervention outcomes. Not all staff interviewed worked at one of the six practices over the entire period of the intervention, which limited their capacity to comment on the earlier stages of implementation. Data was collected at the completion of the pilot study, those interviewed may have forgotten aspects relevant to the early stages of the intervention.

While consideration was given to collecting baseline information from stakeholders about what they expected from enhanced CVD prevention, given the novel nature of the intervention for many of the staff and patients, it was decided that this step would provide little additional information to justify the increased burden on participants. However, the study team did use the NPT constructs in the development of the strategies for the intervention.

The study was strengthened by inclusion of all key workforce groups in general practice, and in particular, by including the patients who were the users of the service. The practices in the study operated using a range of business models and serviced a range of demographic groups. While some of the issues raised were directly related to the predominately fee-for-service funding model of general practice in Australia, many of the findings are relevant to the challenges faced by health systems globally in attempts to improve prevention-orientated primary health care. Finally, use of the NPT provided a rich and detailed framework for analysis and a strong theoretical grounding to the study.

## Conclusion

Despite widespread agreement that increasing CVD prevention activities in primary care is important, progress in reorientating health systems towards prevention-orientated practice has been limited. This study highlights the value of examining the implementation processes at a detailed level, including the experience of all stakeholders, in understanding of the feasibility of a complex intervention being embedded into usual practice. While many barriers will continue to impede the translation of the intervention approach, the study was able to highlight parts of the system that are highly influential to improving CVD prevention and are ready to change. Over time, supporting these areas will increase the capacity of general practice to become a health promoting setting and a true primary health care setting, which is a goal definitely worth pursuing.

## Additional file

Additional file 1: Stakeholder Interview Guide. Stakeholder interview guide questions. (DOCX 18 kb)

## Abbreviations

CVD: Cardiovascular disease
ECCM: Expanded Chronic Care Model
GP: General Practitioner
LA: Lifestyle Advisor
NPT: Normalisation Process Theory
P: Patient
PM: Practice Manager
PN: Practice Nurse
