# Supplementary material for: Implementation of cardiovascular disease prevention in primary health care: enhancing understanding using normalisation process theory
Source: BMC Fam Pract. 2017 Feb 24;18:28. doi: 10.1186/s12875-017-0580-x (PMC5324228; doi:10.1186/s12875-017-0580-x)
Supplement: Additional file 1: — Stakeholder Interview Guide. Stakeholder interview guide questions. (DOCX 18 kb) [file 12875_2017_580_MOESM1_ESM.docx]

**Additional File 1**

**Stakeholder Semi-Structured Interview Guide Questions**

| **Patients** | **General Practitioners** | **Practice Nurses** | **Practice Managers** | **Lifestyle Advisors** |
| --- | --- | --- | --- | --- |
| Overall how did you find the program? | How would you describe your level of commitment to the intervention? | How would you describe your level of commitment to the intervention? | How would you describe your level of commitment to the intervention? | Overall how did you find your involvement in the program? |
| How did you respond to getting the recall letter? | What did you see as the purpose of the intervention? | What did you see as the purpose of the intervention? | What did you see as the purpose of the intervention? | What did you see as the purpose of HeartLink? |
| How is your understanding of your risk of heart disease and how to reduce that risk? | How would you describe your practices commitment to the intervention? | How would you describe your practices commitment to the intervention? | How would you describe your practices commitment to the intervention? | Did the general practices see the purpose of HeartLink? |
| Did your participation in the coaching improve your readiness to change your lifestyle? | Do you think the intervention approach to CVD prevention is compatible with current general practice operation (why /why not)? | Do you think the intervention approach to CVD prevention is compatible with current general practice operation (why /why not)? | Do you think the intervention approach to CVD prevention is compatible with current general practice operation (why /why not)? | Do you think the practices were committed to HeartLink? |
| Did you achieve the goals you set? | Can you comment on whether participants (patients) valued the intervention? | Can you comment on whether participants (patients) valued the intervention? | Can you comment on whether participants (patients) valued the intervention? | Do you think HeartLink is compatible with current general practice operation? |
| What was it about the coaching that helped you do this? | What do you think would have been the key barriers for patients in making changes to reduce their CVD risk (lifestyle change, medication adherence)? | What do you think would have been the key barriers for patients in making changes to reduce their CVD risk (lifestyle change, medication adherence)? | What do you think would have been the key barriers for patients in making changes to reduce their CVD risk (lifestyle change, medication adherence)? | How would you describe your relationship with the general practice you were working with? |
| Do you feel more confident in achieving your lifestyle goals than when you began? | What do you think would have been the key enablers for patients in making changes to reduce their CVD risk? | What do you think would have been the key enablers for patients in making changes to reduce their CVD risk? | What do you think would have been the key enablers for patients in making changes to reduce their CVD risk? | Did the participants see the purpose of HeartLink? |
| Did feel supported by your  •GP  •practice nurse  •lifestyle adviser to set your own lifestyle change goals? | One of the biggest issues was being able to calculate people’s risk (as risk factor wasn’t collected or recorded). Can you comment on why you think this was the case? | One of the biggest issues was being able to calculate people’s risk (as risk factor wasn’t collected or recorded). Can you comment on why you think this was the case? | One of the biggest issues was being able to calculate people’s risk (as risk factor wasn’t collected or recorded). Can you comment on why you think this was the case? | Did the participants value HeartLink? |
| How would you describe your relationship with your general practice? | How confident are you in your skills to assess CVD risk (calculate AR) and support management of that risk? | How confident are you in your skills to assess CVD risk (calculate AR) and support management of that risk? | How confident are you in your skills to assess CVD risk (calculate AR) and support management of that risk? | What were the key barriers for participants in achieving their lifestyle change goals? |
| How would you describe your relationship now with community providers of LMP programs (if you are participating in a program)? | What changes would to the intervention approach based on your experiences? | What changes would to the intervention approach based on your experiences? | What changes would to the intervention approach based on your experiences? | What were the enablers for participants in achieving their lifestyle change goals? |
| Any other comments about your participation in the program | As a community what can we do to improve CVD risk assessment and management (improve heart health)? | As a community what can we do to improve CVD risk assessment and management (improve heart health)? | As a community what can we do to improve CVD risk assessment and management (improve heart health)? | How confident do you now feel in your skill sin supporting people to make lifestyle changes? |
|  |  |  |  | What changes would you make to HeartLink based on your experience? |
